# Artificial intelligence-based screening for amblyopia and its risk factors: comparison with four classic stereovision tests

**DOI:** 10.3389/fmed.2023.1294559

**Published:** 2023-12-22

**Authors:** Zsófia Csizek, Eszter Mikó-Baráth, Anna Budai, Andrew B. Frigyik, Ágota Pusztai, Vanda A. Nemes, László Závori, Diána Fülöp, András Czigler, Kitti Szabó-Guth, Péter Buzás, David P. Piñero, Gábor Jandó

**Affiliations:** ^1^Institute of Physiology, Medical School, University of Pécs, Pécs, Hungary; ^2^Centre for Neuroscience, University of Pécs, Pécs, Hungary; ^3^Institute of Mathematics and Informatics, Faculty of Sciences, University of Pécs, Pécs, Hungary; ^4^Department of Ophthalmology, Medical School, University of Pécs, Pécs, Hungary; ^5^Department of Optics, Pharmacology and Anatomy, University of Alicante, Alicante, Spain

**Keywords:** amblyopia, screening, amblyogenic conditions, artificial intelligence – AI, strabism, cost-effective, ROC (receiver operating characteristic) analysis, amblyopia risk factors

## Abstract

**Introduction:**

The development of costs-effective and sensitive screening solutions to prevent amblyopia and identify its risk factors (strabismus, refractive problems or mixed) is a significant priority of pediatric ophthalmology. The main objective of our study was to compare the classification performance of various vision screening tests, including classic, stereoacuity-based tests (Lang II, TNO, Stereo Fly, and Frisby), and non-stereoacuity-based, low-density static, dynamic, and noisy anaglyphic random dot stereograms. We determined whether the combination of non-stereoacuity-based tests integrated in the simplest artificial intelligence (AI) model could be an alternative method for vision screening.

**Methods:**

Our study, conducted in Spain and Hungary, is a non-experimental, cross-sectional diagnostic test assessment focused on pediatric eye conditions. Using convenience sampling, we enrolled 423 children aged 3.6–14 years, diagnosed with amblyopia, strabismus, or refractive errors, and compared them to age-matched emmetropic controls. Comprehensive pediatric ophthalmologic examinations ascertained diagnoses. Participants used filter glasses for stereovision tests and red-green goggles for an AI-based test over their prescribed glasses. Sensitivity, specificity, and the area under the ROC curve (AUC) were our metrics, with sensitivity being the primary endpoint. AUCs were analyzed using DeLong’s method, and binary classifications (pathologic vs. normal) were evaluated using McNemar’s matched pair and Fisher’s nonparametric tests.

**Results:**

Four non-overlapping groups were studied: (1) amblyopia (*n* = 46), (2) amblyogenic (*n* = 55), (3) non-amblyogenic (*n* = 128), and (4) emmetropic (*n* = 194), and a fifth group that was a combination of the amblyopia and amblyogenic groups. Based on AUCs, the AI combination of non-stereoacuity-based tests showed significantly better performance 0.908, 95% CI: (0.829–0.958) for detecting amblyopia and its risk factors than most classical tests: Lang II: 0.704, (0.648–0.755), Stereo Fly: 0.780, (0.714–0.837), Frisby: 0.754 (0.688–0.812), *p* < 0.02, *n* = 91, DeLong’s method). At the optimum ROC point, McNemar’s test indicated significantly higher sensitivity in accord with AUCs. Moreover, the AI solution had significantly higher sensitivity than TNO (*p* = 0.046, N = 134, Fisher’s test), as well, while the specificity did not differ.

**Discussion:**

The combination of multiple tests utilizing anaglyphic random dot stereograms with varying parameters (density, noise, dynamism) in AI leads to the most advanced and sensitive screening test for identifying amblyopia and amblyogenic conditions compared to all the other tests studied.

## 1 Introduction

Amblyopia (1–3) is a global health problem with an average prevalence of 2.4% (4–12) that is even higher in unscreened populations (13). Early detection is crucial for successful treatment, making regular vision screening in childhood essential (12, 14–16). However, existing literature suggests that screening for amblyopia and its risk factors (or amblyogenic conditions) can be costly, with no effective and really inexpensive screening method currently available (15, 17, 18). The contribution of licensed eye practitioners makes the screening process expensive, and a recent Canadian study demonstrated that universal school screening and optometric examinations have not proven to be cost-effective relative to primary care screening for detecting amblyopia in young children (19). A new high-performance, lay-person-based screening method could considerably reduce costs and make amblyopia screening widely available (15). While stereovision tests that measure stereoacuity have the potential to detect amblyopia and strabismus (20, 21), existing clinical stereovision tests have several limitations, including low sensitivity, particularly in screening situations (22–28).

**Figure 1:**
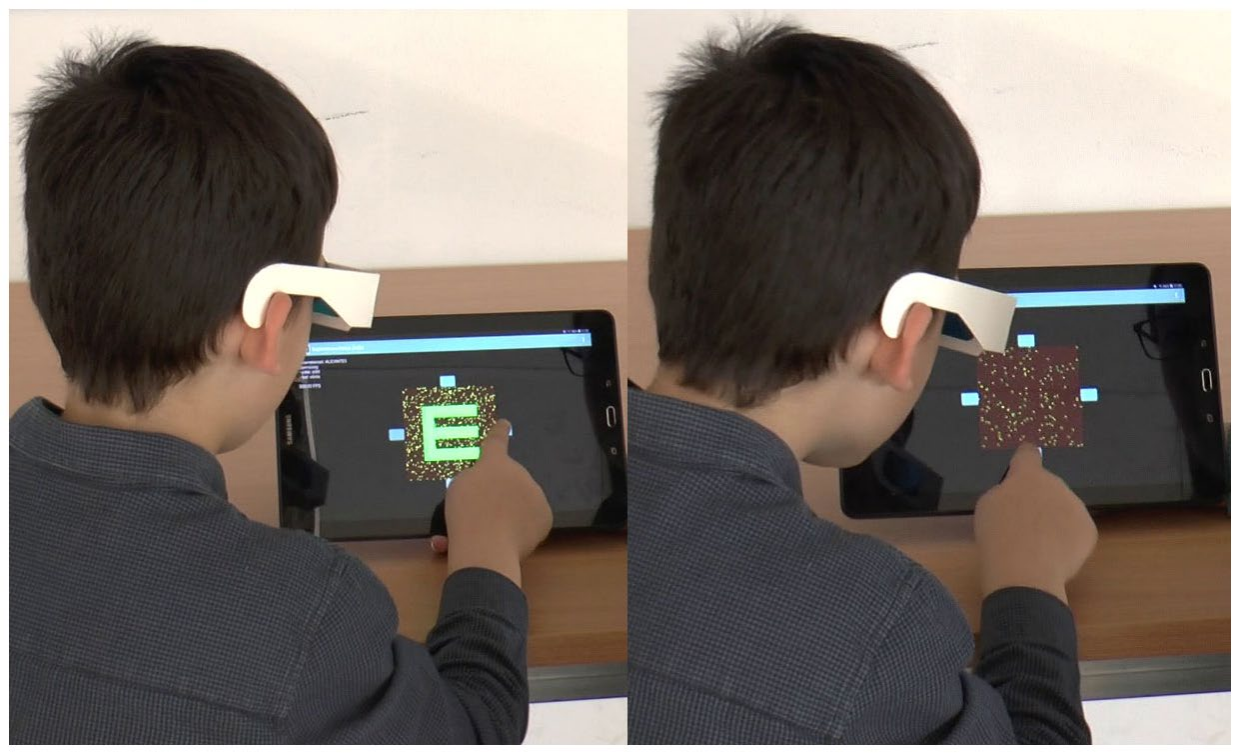
The photo montage is divided into two distinct panels. On the left-hand side, the child is viewing a control display that is universally visible, allowing the child to respond accordingly. Conversely, the right-hand side features an image that is exclusively visible to the child wearing red-green goggles. The child can seamlessly complete the test by pressing the corresponding key, based on the orientation of the Snellen E optotype.

To address these limitations, we developed the EuvisionTab® Stereovision test (ETS), a mobile-based, innovative screening solution for amblyopia (EuvisionTab®, ET, Euvision Ltd., Pécs, Hungary; https://tab.euvision.hu/) (29, 30). The ETS is essentially an anaglyphic random dot stereogram (RDS) generator (Figure 1) with several adjustable parameters, such as frame rate, dot size, dot density, disparity, and noise level, which control the difficulty of binocular perception. Our goal is to create a robust, non-stereoacuity-based stereovision test that meets the criteria of an ideal screening method, including time-efficiency, reproducibility, sensitivity, specificity, ability to make statistically supported decisions, and tolerance to common methodical mistakes. In a previous study, we demonstrated that high-disparity targets embedded in low-density RDS with uncorrelated noise can be a sensitive tool to detect amblyopia and amblyogenic conditions without measuring stereoacuity. However, the low specificity of the noisy stereogram was a limitation (29).

In this study, we aimed to test various settings of the ETS and compare their discrimination performance with the most popular clinical stereovision tests for amblyopia and amblyogenic conditions, which are strictly based on stereoacuity. Furthermore, we will demonstrate how an artificial intelligence-based (AI) algorithm that combines the results of multiple tests with different RDS parameters can dramatically improve specificity without compromising sensitivity.

## 2 Methods

### 2.1 Study design

Our study was a non-experimental cross-sectional diagnostic test study that compared the diagnostic classification of numerous tests with the classification of the ophthalmologist, which was accepted as the “gold standard” (GS). The objective was to identify the best stereovision test for detecting amblyopia and amblyogenic conditions from four classic tests, four novel random dot stereogram tests (ETS), and combinations of the latter by an artificial intelligence trained to maximize sensitivity and specificity (AI-ETS tests). The primary endpoints were sensitivity, specificity, and the area under the receiver operating characteristic (ROC) curve (AUC). We considered sensitivity as the most important measure since failure or delay to discover an amblyopic case reduces the chance of recovery for the child.

In the first phase, participants were tested using classic tests and exploratory versions of the ETS tests, where dot density and noise level of the stereograms were varied, and the four ETS test versions to be included in the second phase were selected. Any test versions with AUC < 0.7 were excluded from the trial. The minimum number of participants for the second phase was estimated based on measurements for the classic tests collected in the first phase of the current study and a previously published paper. (29) MedCalc software was used to estimate a sample size between 24 and 44 for the study group and controls with the target of differentiating (at α = 0.05 and β = 0.2) the average classical test (AUC of 0.75–0.78) from a hypothetical novel test with an AUC of 0.9.

### 2.2 Participants, recruitment, examinations

Participants were recruited from two institutions: Department of Ophthalmology of Vithas Medimar International Hospital of Alicante, Spain (n = 371, between March 2017 and May 2019) and the Department of Ophthalmology, University of Pécs, Hungary (n = 52, between May 2019 and November 2019). The study included 194 healthy emmetropic children aged between 3.8–14 years with a mean age of 7.05 (SD: 2.53) as control subjects, with no ophthalmological or neurological conditions. For the study groups (n = 229, aged 3.6–14 mean age: 7.45 SD: 2.72), children with amblyopia, any type of strabismus, or refractive error were enrolled. Eye conditions were defined according to international guidelines and literature (1, 23, 31, 32) as outlined in Table 1. The demographics of the participants can be found in Table 2. The number of children with and without eye conditions was not significantly different in terms of age (χ2 = 10.1, *p* = 0.122).

**Table 1:** The Definitions of the included eye conditions.

Name	Definition
Amblyopia	Reduced best corrected visual acuity (0.2 or more logMAR lines of interocular difference) regardless of the origin (anisometropia, strabismus or mixed)
Anisometropia	One or more diopters difference in refractive error between the two eyes
Strabismus	Abnormal alignment of the eyes, including esotropia, exotropia, convergence insufficiency, microesotropia, accommodative esotropia, decompensated phoria, hypertropia and intermittent exotropia
Significant hyperopia	Hyperopia equal or exceeding 2.5 D under cycloplegia
Amblyogenic conditions	Strabismus, anisometropia and significant hyperopia (children having amblyopia are not included)
Nonamblyogenic	Myopia, astigmia and hyperopia less than 2.5 D (children having amblyopia or/and amblyogenic condition are not included)

D: diopters; logMAR: logarithm of minimum angle of resolution. Definition of amblyopia and preamblyopic conditions are based on the international literature.

**Table 2:** Demographic distribution of participants.

Age at screening (yr)	Eye condition	Control	N	% of total
3.5-4	4	1	5	1.18
4-5	45	59	104	24.59
5-6	28	24	52	12.29
6-7	40	27	67	15.84
7-8	31	22	53	12.53
8-9	19	21	40	9.46
9-15	62	40	102	24.11
Total	229	194	423	100.00
Female			193	46.63
Male			230	54.37

The study was approved by the local ethics committees (Alicante: UA-2017-03-20, Pécs: 6301/2016) and was conducted in accordance with the Helsinki Declaration. Written informed consent was obtained from all parents or legal guardians after they were fully informed of the nature, course, advantages, and disadvantages of the investigation, both in oral and written forms.

The diagnostic classification was determined through a comprehensive eye examination conducted by licensed eye care professionals. The examination included monocular best-corrected visual acuity measurement using calibrated Snellen charts, objective and subjective refraction with and without cycloplegia, eye movement examination, cover test, Brückner test, Hirschberg test, Worth’s four dot test, a 4 diopter prism test to detect microtropia, and a monocular estimate method retinoscopy to evaluate the accommodative response.

### 2.3 Stereovision tests and procedures

In this study, the newly developed non-stereoacuity-based stereovision test (ETS) was used and its results were compared with those of four traditional, stereoacuity-based clinical stereovision tests (Lang II, TNO, Frisby, and Stereo Fly or Titmus Fly). (Table 3).

**Table 3:** Summary of stereovision tests.

Test name	Stimuli	Channel separation	Type of stereotest	Producer	Viewing distance (cm)	Possible results	Number of participants tested
Classic tests							
Lang II	star, elephant, car, moon	panographic	global; random dot	Lang Stereotest AG, Forch, langstereotest.com	40	>1000”* 600” 400” 200”	423
TNO	Plate V-VI, „pancake”	anaglyphic	global; random dot	Lameris Ootech BV, ootech.nl	40	>1000” 480” 240” 120” 60”	385
Frisby	circles	not needed	global; real depth	Frisby StereotestTM, frisbystereotest.co.uk	40	>1000” 340” 170” 85”	265
Stereo Fly	circles	polarization method	local; contour stereogram	Stereo Optical Company, INC., stereooptical.com	40	>1000” 800” 400” 200” 140” 100” 80” 60” 50” 40”	249
ETS SRDS 8 DRDS 1 DRSD 0.7 DRDS 1+noise	Snellen E	anaglyphic	global; random dot	Euvision Ltd., Pécs, Hungary	25-30	0-5/5 0-5/5 0-5/5 0-5/5	254 130 130 254
AI-ETS sum w aw	Snellen E	anaglyphic	global; random dot	Euvision Ltd., Pécs, Hungary	25-30	0-20/20 0-20/20 0-20/20	130 130 130

“: sec of arc; ETS: EuvisionTab® Stereovision test module; NC: no correction; WC: with correction; SRDS 8: 8% density static test; DRDS 1: 1% density dynamic test; DRDS 0.7: 0.7% density dynamic test; DRDS 1+noise: 1% density dynamic test with 0.5% binocularly uncorrelated noise; AI-ETS: Artificial intelligence-based ETS tests; sum: equally weighted sum of the four ETS-tests; w: ‘weight’, optimized weight for amblyopia; aw: ‘average weight’, optimized weight for all pathologic conditions, *>1000” refers to lack of stereopsis.

The ETS was performed using a 10.1-inch tablet (two types were used: 1. Samsung Galaxy Tab A (2016) 2. BQ Aquaris M10) at a viewing distance of 25–30 cm, and the patient responses were registered via input keys (Figure 1). The dot size was 420″, while the disparity of the stimuli was 840″ at 25 cm viewing distance. The size of the Snellen E was approximately 2°. The procedure for the ETS was described in detail by Budai et al. (29).

In the four non-stereoacuity-based ETSs, three parameters of the RDS were varied to create different levels of difficulty: Firstly, the RDSs were either static (SRDS) or dynamic (DRDS). In the dynamic stimuli, the random dot matrices were refreshed at 30 Hz. Secondly, the density was varied, which refers to the proportion of bright and dark dots in the RDS. In this study, three combinations of dynamism and density were used: 8% static (SRDS 8), 1% dynamic (DRDS 1), and 0.7% dynamic (DRDS 0.7). Finally, the noise level was varied, which represents the proportion of binocularly uncorrelated dots added to the stereogram. One condition included in the test was where 0.5% uncorrelated noise was added to the DRDS 1 condition (DRDS 1 + noise) (Table 3). In each ETS testing session, 24 test stimuli were presented in the following sequence: 1 repetition (x) of monocular control - 5 x SRDS 8–1 x monocular control - 5 x DRDS 1–1 x monocular control – 5 x DRDS 0.7–1 x monocular control – 5 x DRDS 1 + noise. Each participant was tested with a stereovision test only once, and retests were not performed. Not all participants were tested with all stereopsis tests. Children with a prescription for refractive glasses underwent the ETS both with (WC) and without (NC) refractive corrections.

To reduce the examination time, a relatively small number of images were presented for each type of ETS test. As a result, each examination formed a Bernoulli trial. For a trial to be considered successful, the binomial cumulative probability of false responses had to be less than 0.05. Each participant was presented with only five stereograms, so to pass the test, they had to correctly identify at least three of them, even if the ROC analysis suggested an optimum value of less than three (29). For the combined tests, the Bernoulli criteria were always met at the optimum ROC point, which was around 12–15 correct responses out of 20.

The same experienced examiner performed the stereotests at both institutions. The four traditional stereovision tests were administered under daylight conditions, whereas the ETS tests were conducted in a dark room with participants wearing red-green goggles.

### 2.4 Application of artificial intelligence

**Figure 2:**
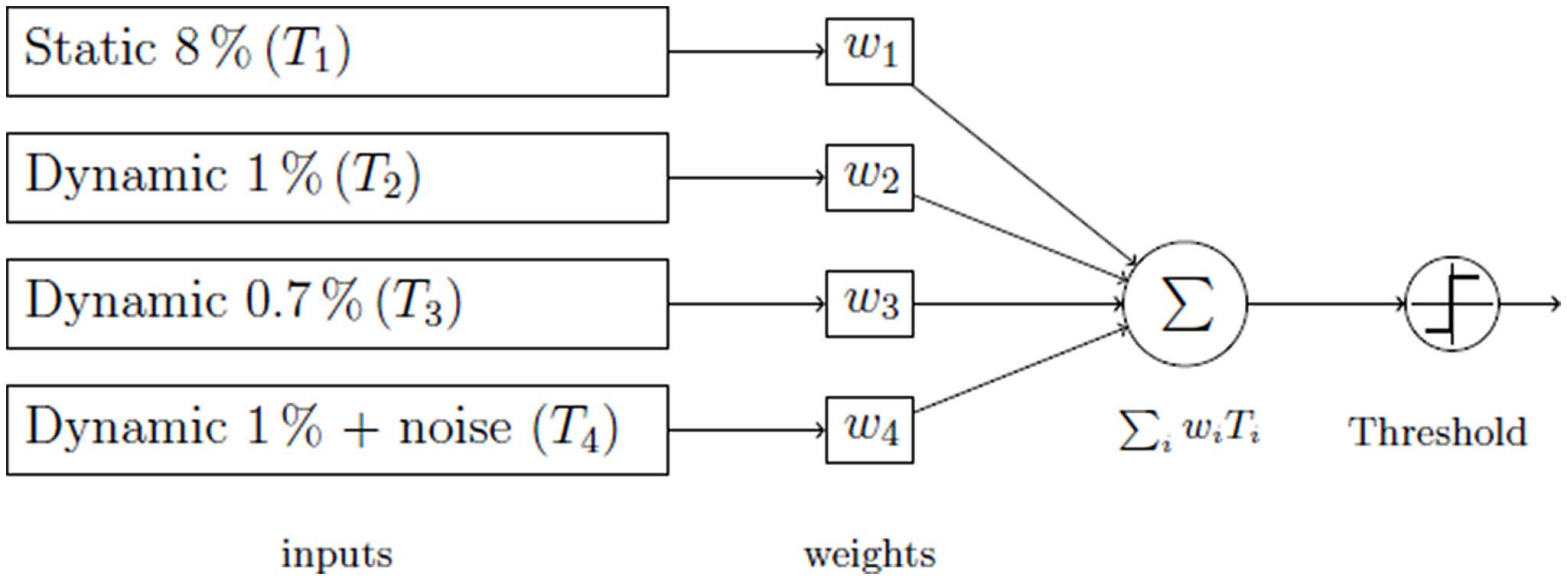
The Perceptron neural net utilized for decision-making.

In order to improve the accuracy of ETS screening, we used a weighted combination (AI-ETS) of the results from the four tests to create a new metric. To prevent overfitting and maintain generalization in AI solutions, we chose a straightforward model: the Perceptron model (33), a simple linear integrator (Figure 2).

To make a decision, we combined the results of all tests and created a variable based on ETS scores. We used three different methods for this combination: 1) Equal weight sum (simple addition), 2) Average weight sum with optimized weights for all study groups, and 3) Weight sum with optimized weights specifically for amblyopia. We used a least-square algorithm to determine the weights that minimized the deviation from 100% sensitivity and specificity.

#### 2.4.1 The artificial intelligence model

The objective with this model is to ascertain optimal weights, often referred to as mixture parameters, for the Perceptron neural network. The goal was to minimize the discrepancy between the actual network outcome and a target value. The inputs to the model comprised results from four distinct tests: one static and three dynamic. The aim was to identify a set of weights, denoted as *w_1_, w_2_, w_3_*, and *w_4_*, ensuring the cumulative result of these tests surpasses that of any individual test. The combination of these tests dictated if the subject has passed or failed. We collated these outcomes within a contingency table and calculated the sensitivity and specificity of this test amalgamation. The squared deviation of these parameters from the perfect score of 1 served as our error function to minimize.

#### 2.4.2 Optimization of the weights

Initially, for simplicity, we employed the Simulated Annealing method (34, 35), which we implemented in a custom-made MATLAB program. This iterative process ultimately provided us with a set of optimal weights, although it is noteworthy that this optimum may not always be global.

While the Simulated Annealing optimization technique was suitable for training a Perceptron model on an individual basis, its limitations preclude a detailed examination of the Perceptron’s full spectrum of opportunities and the extent to which individual inputs contribute to its efficacy. To gain a deeper understanding of the Perceptron model’s overall performance and the role of each input variable, it was necessary to employ additional, faster methods and tools. We opted to use MATLAB’s Neural Network Toolbox, which offers a wide spectrum of transfer functions and weight optimization methods.

In our study, we also included cross-validation to ensure that our model’s generalizability is robust across different datasets. This process allows us to assess the consistency and reliability of the Perceptron.

#### 2.4.3 Simulated annealing

The Simulated Annealing algorithm, rooted in Thermodynamics yet widely applicable, was employed to tackle the aforementioned problem. Imagine a set of configurations symbolizing potential solutions for a given problem. Let us assume a function *F* is defined over this configuration space, which we desire to either minimize or maximize. For this discussion, we’ll focus on minimizing *F*. Let $\bar{\xi }$ represent the present configuration and *T* symbolize the system’s “temperature,” influenced by the cooling rate. The following steps delineate the process to pinpoint the optimal configuration:

1. Derive a fresh configuration *ξ* that’s in proximity to the existing one.
2. Decrease the temperature *T* in line with the cooling procedure.
3. Determine the differential:
$$F\left(\bar{\xi }\right)-F\left(\xi \right).$$
4. If this difference is positive, indicating the function *F* at the new configuration is less than its predecessor, the new configuration is retained, and the old one is discarded.
5. Conversely, if this difference is negative, the new sample is not instantly rejected. Instead, it’s accepted as the new configuration based on a probability defined by the Boltzmann factor:
$$\exp\left(\frac{F\left(\bar{\xi }\right)-F\left(\xi \right)}{T}\right).$$
6. This loop continues until the cooling process reaches a stopping point.

#### 2.4.4 Assessing input significance and generalization of the Perceptron

**Figure 3:**
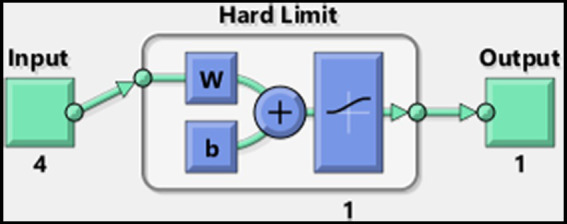
MATLAB’s Perceptron neural network architecture for assessment of input significance and generalization.

The MATLAB Neural Network Toolbox offers a graphical view of the currently applied network. The architecture of our Perceptron model is depicted in Figure 3.

In pursuit of the swiftest convergence, we replaced the initially employed network, which had a linear output, with one utilizing a logistic sigmoid (logsig) transfer function. This adjustment enabled the use of more rapid learning algorithms. The Levenberg–Marquardt algorithm was implemented for training, significantly accelerating convergence. The weights and bias values were randomly initialized within the range of 0 to 1. To enhance the training process and promote convergence, a homogeneous noise margin of approximately ±3% was introduced into the dataset, a technique commonly used to prevent overfitting and to promote generalization within the model. For dataset preparation, stereovision test results with and without refractive correction were merged to create a unified training dataset consisting of 182 four-dimensional vectors.

To evaluate the impact of each input on the model’s performance, we tested all possible mathematical combinations of the four inputs, resulting in 15 different scenarios: individual tests (4), all pairs (6), triplets (4), and the complete set of four tests (1). Training to assess input significance was conducted on the complete dataset, while a randomly partitioned subset was used to test for generalization.

To ensure the robustness of our model and rule out overfitting as a potential bias in performance, we employed the random division technique for dataset partitioning. Overfitting occurs when a model learns the details and noise in the training data to the extent that it negatively impacts the model’s performance on new data. We tackled this by randomly dividing the dataset into two parts: 75% for training and 25% for testing.

To thoroughly investigate the convergence consistency of our model, hundred independent training sessions were conducted, each involving the reinitialization of the model’s parameters and random repartitioning of the training and validation sets for each session. After each session, the AUC was calculated. Analyzing the AUC values across all runs allowed us to quantify the variability and stability of the model’s performance and to statistically compare results. The mean and standard deviation of the AUC scores offered insight into the convergence behavior of our training process, enabling an assessment of both the input significance and the performance differences between the validation and training sets across multiple initializations and training cycles.

### 2.5 Statistical analysis

Data processing was performed using MATLAB 2018b (The MathWorks, Inc., Natick, Massachusetts, United States), while for ROC analysis MedCalc® Statistical Software version 20.211 (MedCalc Software Ltd, Ostend, Belgium; https://www.medcalc.org; 2023) was used. To compare the performance of classic stereovision tests and various versions of the ETS (Table 4), the following methods were applied:

1. AUCs were calculated and compared using DeLong’s method as implemented in MedCalc, which is designed for multiple comparisons (36).
2. Sensitivities and specificities were compared at the optimum ROC point.
  After binary classification (pathologic vs. normal):
3. McNemar’s matched pair comparison was used to determine significant differences between classic and AI-based tests.
4. Fisher’s exact test was used to compare the sensitivity and specificity of the AI-aw WC and TNO tests.

**Table 4:** Nomenclature of the stereovision tests compared in this study.

Screening test	Refractive correction	Dynamic	Density of RDS (%)
Classic tests			
Lang II	Yes	No	50
TNO	Yes	No	Not specified
Stereo Fly	Yes	No	Contour
Frisby	Yes	No	Patterned
Single ETSs			
SRDS 8 NC	No	No	8
DRDS 1 NC	No	Yes	1
DRDS 0.7 NC	No	Yes	0.7
DRDS 1+noise NC	No	Yes	1
Artificial intelligence-based tests (AI-ETS)			
AI-sum NC (equal weighted sum)	No	Mixed	Various
AI-w NC (weighted for amblyopia)	No	Mixed	Various
AI-aw NC (weighted for all conditions)	No	Mixed	Various
Single ETSs			
SRDS 8 WC	Yes	No	8
DRDS 1 WC	Yes	Yes	1
DRDS 0.7 WC	Yes	Yes	0.7
DRDS 1 + noise WC	Yes	Yes	1
Artificial intelligence-based tests			
AI-sum WC (equal weighted sum)	Yes	Mixed	Various
AI-w WC (weighted for amblyopia)	Yes	Mixed	Various
AI-aw WC (weighted for all conditions)	Yes	Mixed	Various

“: sec of arc; ETS: EuvisionTab® Stereovision test module; NC: no correction; WC: with correction; SRDS 8: 8% density static test; DRDS 1: 1% density dynamic test; DRDS 0.7: 0.7% density dynamic test; DRDS 1+noise: 1% density dynamic test with 0.5% binocularly uncorrelated noise; AI-ETS: Artificial intelligence-based ETS tests; sum: equally weighed sum of the four ETS-tests; w: ‘weight’, optimized weight for amblyopia; aw: ‘average weight’, optimized weight for all pathologic conditions.

To control for type I and type II statistical errors due to multiple pairwise comparisons, we applied Bonferroni’s or Benjamini-Hochberg’s (37) methods. Further details on these statistical tests can be found in the Supplementary Methods.

## 3 Results

### 3.1 Characteristics of participants

We enrolled 229 participants with a range of eye conditions (Table 2). These participants were segmented into four non-overlapping groups, with each individual potentially having more than one underlying diagnosis. The amblyopia group (n = 46) consisted of 17 children with anisometropic, 12 with strabismic, and 17 with mixed amblyopia. The amblyogenic condition group (n = 55) included 30 individuals with strabismus, 19 with anisometropia, and 35 with hyperopia of any degree. The nonamblyogenic condition group (n = 128) comprised 23 children with myopia, 92 with non-significant hyperopia, and 53 with astigmatism. The control group was made up of 194 emmetropic participants. Furthermore, a fifth joint “amblyopia + amblyogenic” group was created to identify amblyopia as well as amblyogenic conditions. Stereovision tests, along with the variations of the ETSs, are summarized in Table 3. Table 5 outlines the distribution of participants in each study group and the control group who underwent each stereovision test.

**Table 5:** Participant count by group for various stereovision tests.

Screening test	Amblyopia	Amblyogenic	Nonamblyogenic	Control	Total
SRDS 8 NC	28	36	66	124	254
DRDS 1 NC	28	36	66	124	254
DRDS 0.7 NC	23	23	39	45	130
DRDS 1+noise NC	23	23	39	45	130
AI-sum NC	23	23	39	45	130
AI-w NC	23	23	39	45	130
AI-aw NC	23	23	39	45	130
SRDS 8 WC	35	39	106	124	304
DRDS 1 WC	35	39	106	124	304
DRDS 0.7 WC	23	23	39	45	130
DRDS 1+noise WC	23	23	39	45	130
AI-sum WC	23	23	39	45	130
AI-w WC	23	23	39	45	130
AI-aw WC	23	23	39	45	130
Lang II	46	55	128	194	423
TNO	41	47	114	183	385
Stereo Fly	34	40	60	115	249
Frisby	38	43	63	119	263
Number of participants examined with all tests	23	23	39	45	130
Number of participants in the group	46	55	128	194	423

NC: no correction; WC: with correction; SRDS 8: 8% density static test; DRDS 1: 1% density dynamic test; DRDS 0.7: 0.7% density dynamic test; DRDS 1+noise: 1% density dynamic test with 0.5% binocularly uncorrelated noise; AI-ETS: Artificial intelligence-based ETS tests; sum: equally weighted sum of the four ETS-tests; w: ‘weight’, optimized weight for amblyopia; aw: ‘average weight’, optimized weight for all pathologic conditions.

### 3.2 Classification performance: area under the ROC curve

We assessed the efficacy of various stereovision tests in differentiating between individuals with diverse eye conditions and the emmetropic control group. This assessment was carried out by calculating the AUCs and using DeLong’s method for pairwise comparisons (Tables 6, 7). All tests outperformed a random classifier in identifying amblyopia or amblyogenic conditions. Nevertheless, pairwise comparisons revealed that for the amblyogenic and joint amblyopia+amblyogenic group, the optimized AI-ETSs versions (i.e., AI-w WC, AI-aw WC) yielded higher AUCs than classic tests, except for the TNO (Table 6). These differences were statistically significant for all the above-mentioned pairs (Table 7) (*n* = 91).

**Table 6:** Receiver operating characteristic curve analysis of the stereo tests: AUC values with 95% confidence intervals.

Test name	Amblyopia	Amblyogenic	Nonamblyogenic	Amblyopia+ Amblyogenic
SRDS 8 NC	0.910 (0.852-0.950)	0.693 (0.615-0.763)	0.508 (0.435-0.581)	0.788 (0.722-0.844)
DRDS 1 NC	0.918 (0.862-0.956)	0.685 (0.607-0.756)	0.525 (0.452-0.598)	0.787 (0.721-0.843)
DRDS 0.7 NC	0.976 (0.906-0.998)	0.856 (0.749-0.929)	0.558 (0.445-0.666)	0.916 (0.839-0.964)
DRDS 1+noise NC	0.914 (0.821-0.969)	0.829 (0.718-0.909)	0.599 (0.486-0.705)	0.872 (0.785-0.933)
AI-sum NC	0.995 (0.937-1.000)	0.876 (0.774-0.944)	0.606 (0.494-0.711)	0.936 (0.864-0.976)
AI-w NC	0.996 (0.940-1.000)	0.867 (0.762-0.937)	0.604 (0.491-0.709)	0.931 (0.859-0.974)
AI-aw NC	0.996 (0.940-1.000)	0.865 (0.760-0.936)	0.614 (0.501-0.718)	0.930 (0.857-0.973)
SRDS 8 WC	0.889 (0.830-0.933)	0.641 (0.563-0.715)	0.513 (0.447-0.580)	0.759 (0.693-0.816)
DRDS 1 WC	0.853 (0.788-0.904)	0.629 (0.550-0.703)	0.511 (0.445-0.578)	0.735 (0.667-0.795)
DRDS 0.7 WC	0.934 (0.846-0.980)	0.689 (0.566-0.796)	0.576 (0.464-0.684)	0.812 (0.716-0.886)
DRDS 1+noise WC	0.919 (0.827-0.971)	0.671 (0.547-0.781)	0.536 (0.423-0.645)	0.795 (0.698-0.873)
AI-sum WC	0.972 (0.901-0.997)	0.805 (0.718-0.909)	0.596 (0.483-0.702)	0.889 (0.806-0.945)
AI-w WC	0.971 (0.898-0.996)	0.830 (0.719-0.910)	0.613 (0.501-0.718)	0.900 (0.819-0.953)
AI-aw WC	0.976 (0.906-0.998)	0.840 (0.731-0.917)	0.611 (0.499-0.716)	0.908 (0.829-0.958)
Lang II	0.822 (0.768-0.869)	0.604 (0.541-0.666)	0.522 (0.466-0.578)	0.704 (0.648-0.755)
TNO	0.953 (0.916-0.977)	0.742 (0.680-0.797)	0.603 (0.544-0.659)	0.840 (0.791-0.882)
Stereo Fly	0.926 (0.871-0.962)	0.656 (0.576-0.731)	0.585 (0.508-0.659)	0.780 (0.714-0.837)
Frisby	0.852 (0.786-0.903)	0.668 (0.590-0.740)	0.528 (0.453-0.603)	0.754 (0.688-0.812)

AUC: area under the ROC curve; for all conditions and across all classifiers, the AUC was significantly greater than 0.5, with the exception of non-amblyogenic conditions; NC: no correction; WC: with correction; SRDS 8: 8% density static test; DRDS 1: 1% density dynamic test; DRDS 0.7: 0.7% density dynamic test; DRDS 1+noise: 1% density dynamic test with 0.5% binocularly uncorrelated noise; AI-ETS: artificial intelligence-based ETS tests; sum: equally weighted sum of the four ETS-tests; w: ‘weight’, optimized weight for amblyopia; aw: ‘average weight’, optimized weight for all pathologic conditions.

**Table 7:** Pairwise comparison of classic and ETS tests for AUC values.

Amblyopia	Lang II	TNO	Stereo Fly	Frisby
SRDS 8 WC	0.404	0.707	0.745	0.210
DRDS 1 WC	0.381	0.737	0.733	0.246
DRDS 0.7 WC	0.134	0.627	0.748	0.271
DRDS 1+noise WC	0.659	0.318	0.992	0.457
AI-sum WC	**0.041**	0.458	0.240	**0.030**
AI-w WC	0.067	0.540	0.288	**0.034**
AI-aw WC	**0.042**	0.391	0.215	**0.023**
Amblyogenic conditions				
SRDS 8 WC	0.182	0.715	0.399	0.313
DRDS 1 WC	0.484	0.564	0.463	0.518
DRDS 0.7 WC	0.492	0.101	0.728	0.800
DRDS 1+noise WC	0.875	0.082	0.923	0.982
AI-sum WC	0.086	0.924	0.096	0.064
AI-w WC	**0.038**	0.556	**0.046**	**0.028**
AI-aw WC	**0.018**	0.409	**0.038**	**0.014**
Ambylopia+Amblyogenic conditions				
SRDS 8 WC	0.124	0.638	0.371	0.141
DRDS 1 WC	0.327	0.534	0.435	0.266
DRDS 0.7 WC	0.205	0.115	0.653	0.443
DRDS 1+noise WC	0.954	0.074	0.941	0.672
AI-sum WC	**0.015**	0.684	**0.047**	**0.008**
AI-w WC	**0.008**	0.447	**0.027**	**0.004**
AI-aw WC	**0.003**	0.289	**0.018**	**0.001**

Numbers represent p-values, level of significance was determined at p=0.05. *p*-values less than 0.05 are in bold. AUC: area under the ROC curve. ETS: EuvisionTab® Stereovision test module; NC: no correction; WC: with correction; SRDS 8: 8% density static test; DRDS 1: 1% density dynamic test; DRDS 0.7: 0.7% density dynamic test; DRDS 1+noise: 1% density dynamic test with 0.5% binocularly uncorrelated noise; AI-ETS: artificial intelligence-based ETS tests; sum: equally weighted sum of the four ETS-tests; w: ‘weight’, optimized weight for amblyopia; aw: ‘average weight’, optimized weight for all pathologic conditions.

### 3.3 Sensitivities and specificities at the optimum ROC point

In the subsequent phase of our statistical analysis, binary classification (pathologic vs. normal) was conducted at the optimum ROC point. For each stereovision test, we calculated sensitivities for every study group and specificities for the control group, as detailed in Table 8.

**Table 8:** Sensitivity and specificity at optimal ROC points for each condition and test.

Test name	Amblyopia	Amblyogenic	Nonamblyogenic	Amblyopia+Amblyogenic	Specificity
SRDS 8 NC	0.86 (0.67-0.96)	0.47 (0.30-0.65)	0.08(0.03-0.17)	0.64 (0.51-0.76)	0.98 (0.93-0.99)
DRDS 1 NC	0.89 (0.72-0.98)	0.44 (0.28-0.62)	0.15(0.08-0.26)	0.64 (0.51-0.76)	0.88 (0.81-0.93)
DRDS 0.7 NC	0.87 (0.66-0.97)	0.52 (0.31-0.73)	0.21(0.09-0.36)	0.70 (0.54-0.82)	0.96 (0.85-0.99)
DRDS 1+noise NC	0.83 (0.61-0.95)	0.57 (0.34-0.77)	0.21(0.09-0.36)	0.70 (0.54-0.82)	0.87 (0.73-0.95)
AI-sum NC	1.00 (0.85-1.00)	0.65 (0.43-0.84)	0.33(0.19-0.50)	0.83 (0.69-0.92)	0.91 (0.79-0.98)
AI-w NC	1.00 (0.85-1.00)	0.61 (0.39-0.80)	0.13(0.04-0.27)	0.80 (0.66-0.91)	0.98 (0.88-1.00)
AI-aw NC	1.00 (0.85-1.00)	0.70 (0.47-0.87)	0.26(0.13-0.42)	0.85 (0.71-0.94)	0.89 (0.76-0.96)
SRDS 8 WC	0.77 (0.60-0.90)	0.36 (0.21-0.53)	0.11(0.06-0.19)	0.55 (0.43-0.67)	0.98 (0.93-0.99)
DRDS 1 WC	0.74 (0.57-0.88)	0.38 (0.23-0.55)	0.20(0.13-0.29)	0.55 (0.43-0.67)	0.88 (0.81-0.93)
DRDS 0.7 WC	0.83 (0.61-0.95)	0.39 (0.20-0.61)	0.21(0.09-0.36)	0.61 (0.45-0.75)	0.96 (0.85-0.99)
DRDS 1+noise WC	0.83 (0.61-0.95)	0.35 (0.16-0.57)	0.21(0.09-0.36)	0.59 (0.43-0.73)	0.87 (0.73-0.95)
AI-sum WC	0.96 (0.78-1.00)	0.43 (0.23-0.66)	0.28(0.15-0.45)	0.70 (0.54-0.82)	0.91 (0.79-0.98)
AI-w WC	0.96 (0.78-1.00)	0.43 (0.23-0.66)	0.10(0.03-0.24)	0.70 (0.54-0.82)	0.98 (0.88-1.00)
AI-aw WC	0.96 (0.78-1.00)	0.70 (0.47-0.87)	0.23(0.11-0.39)	0.83 (0.69-0.92)	0.89 (0.76-0.96)
Lang II	0.65 (0.50-0.79)	0.22 (0.12-0.35)	0.05(0.02-0.11)	0.42 (0.32-0.52)	0.99 (0.96-1.00)
TNO	0.88 (0.74-0.96)	0.47 (0.32-0.62)	0.20(0.13-0.29)	0.66 (0.55-0.76)	0.91 (0.86-0.95)
Stereo Fly	0.85 (0.69-0.95)	0.30 (0.17-0.47)	0.17(0.08-0.29)	0.55 (0.43-0.67)	0.97 (0.93-0.99)
Frisby	0.76 (0.60-0.89)	0.44 (0.29-0.60)	0.19(0.10-0.31)	0.59 (0.48-0.70)	0.86 (0.78-0.91)

Values in brackets represent 95% confidence intervals. ROC: receiver operating characteristic, NC: no correction; WC: with correction; SRDS 8: 8% density static test; DRDS 1: 1% density dynamic test; DRDS 0.7: 0.7% density dynamic test; DRDS 1+noise: 1% density dynamic test with 0.5% binocularly uncorrelated noise; AI-ETS: artificial intelligence-based ETS tests; sum: equally weighted sum of the four ETS-tests; w: ‘weight’, optimized weight for amblyopia; aw: ‘average weight’, optimized weight for all pathologic conditions.

Every test evaluated in this study exhibited a specificity of at least 86%. However, the sensitivity varied widely among the tests, influenced by the type of eye condition. Drawing from average sensitivity metrics, the AI tests surpassed both individual tests and traditional stereoacuity-based clinical stereovision evaluations in terms of sensitivity, consistent with the AUC data. Notably, the AI-aw test demonstrated the highest sensitivity for both amblyopia and amblyogenic conditions. In contrast, no tests presented a significant sensitivity for nonamblyogenic conditions. We also observed that introducing refractive correction enhanced visual performance for amblyogenic conditions. This adjustment resulted in diminished sensitivities in the WC group of tests when juxtaposed with the NC group.

### 3.4 Comparison of binary classification

We also sought to determine whether the observed significant differences in AUC figures translated into significant variations in sensitivity following binary classification. To test the null hypothesis—that the performance of classic tests does not differ from that of AI-aw WC employed McNemar’s exact pairwise statistical comparison. Our analysis indicated that, for the combined amblyopia and amblyogenic study group, AI-aw WC consistently outperformed all classic tests, with the sole exception of the TNO. Specifically, the comparisons between AI-aw WC and Frisby, Lang II, Stereo Fly and TNO tests resulted in differences, with *p*-values of 0.0117, 0.0129, 0.0129, and 0.508, respectively, (n = 46). To account for multiple comparisons, we applied the Benjamini-Hochberg method for controlling the false discovery rate, and found that the interpretation of significance did not change. Furthermore, no significant difference was observed in the emmetropic group.

While we could not utilize all the data for the matched pair McNemar comparison, the sensitivity figures and their 95% confidence intervals suggested a potentially significant difference between TNO [0.66 (95%CI: 0.55–0.76)] and AI-aw WC [0.83 (95%CI: 0.69–0.92)]. Recognizing the uneven sample sizes of the compared groups, we employed the Fisher’s exact non-parametric test, which accommodates such discrepancies. This analysis AI-aw WC’s. superior performance, highlighting a significant difference in sensitivity relative to the TNO. The Fisher’s exact test yielded a value of p = 0.046 for the sensitivity comparison (*n* = 134), while the specificity comparison for the emmetropic group resulted in a value of p = 0.575 (*n* = 228).

For further evidence and in-depth analysis of the data, please also refer to Supplementary Table S1 and Supplementary Figures S1, S2 in the supplementary material.

### 3.5 Modeling performance and convergence

The modifications to the derivable output function, combined with the adoption of the Levenberg–Marquardt training algorithm, significantly accelerated convergence of the Perceptron. This enhancement facilitated efficient testing, even with hundreds of random initializations and repeated training sessions.

#### 3.5.1 Role of inputs to the Perceptron

**Figure 4:**
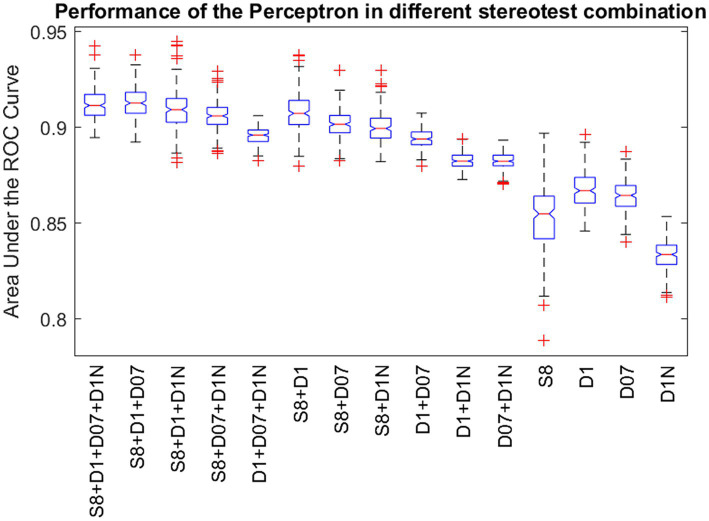
Performance of the Perceptron in different stereovision test combinations. Standard boxplot shows the distribution of the AUCs after 100 repetitions of reinitialization and convergence for single tests, pairs, triplets and all four input variations. Boxes are bounded by the first and third quartiles, red lines in the boxes show the medians and whiskers show the lowest and highest data points within 1.5 times the interquartile range to the median. Red crosses represent outliers. S8: Static random dot stereogram with 8% density, D1: Dynamic random dot stereogram with 1% density, D07: random dot stereogram with 0.7% density, D1N: Dynamic random dot stereogram with 1% density including 0.5% uncorrelated noise.

**Figure 5:**
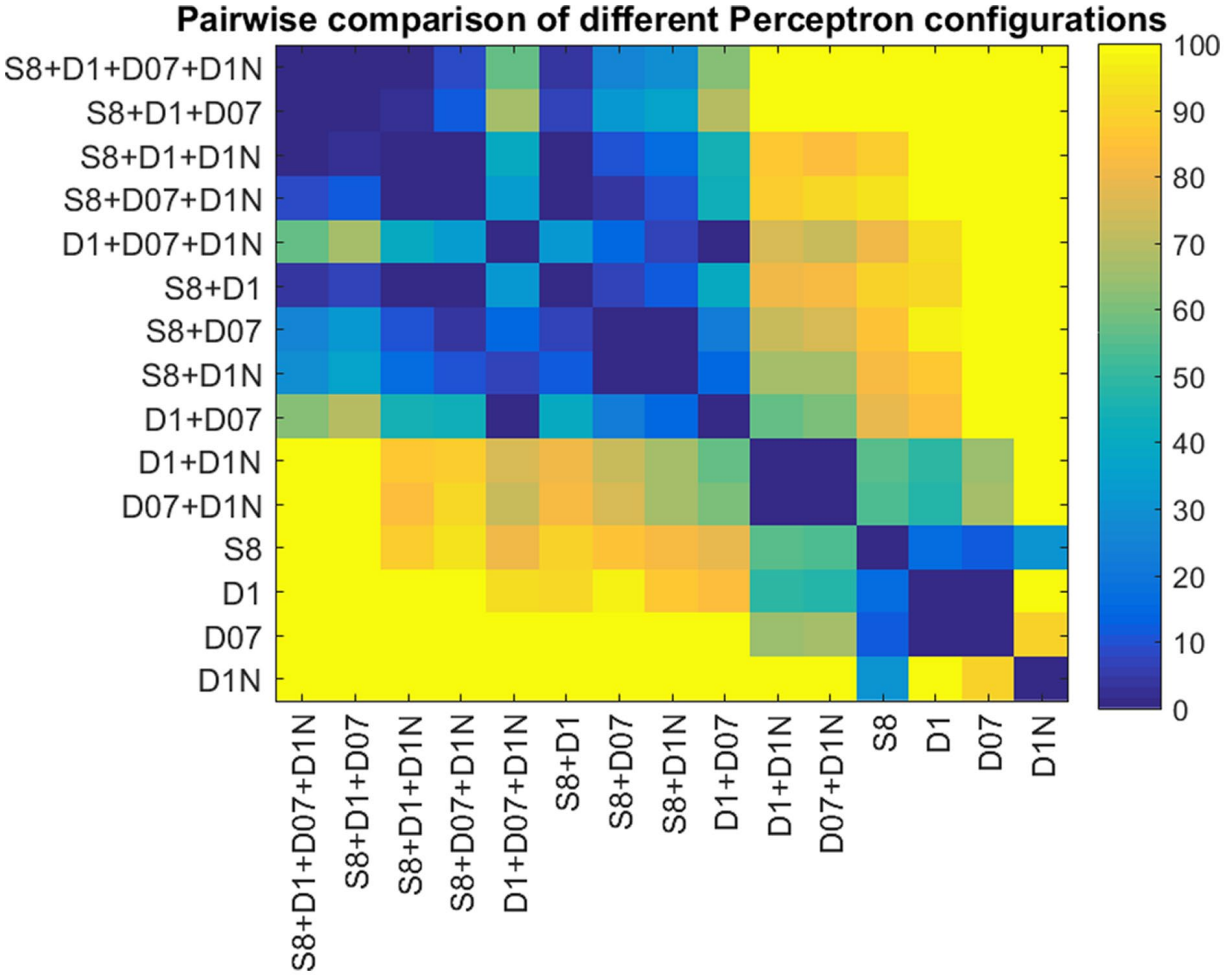
Pairwise comparison of the AUCs for different Perceptron configurations. The color codes represent the *p*-values obtained from pairwise Student *t*-tests. *p*-values are coded as the negative logarithm of their magnitudes.

Figure 4 shows a boxplot of our results from all possible combinations of inputs, including single tests, pairs, triplets, and all four inputs simultaneously. It is evident that single test performance is inferior compared to combinations involving two or more tests. Results are particularly superior when static and at least one dynamic test are combined. The noisy stereogram appears to contribute the least efficiently to the overall performance, as its exclusion does not significantly diminish the performance variable (AUC). Figure 5 displays the pairwise comparison of AUCs for all 15 combinations of inputs. After applying Bonferroni’s correction, which adjusted the significance threshold by a factor of 15×15, the negative logarithm of the adjusted *p*-values was color-coded for visualization in the figure.

#### 3.5.2 Generalization ability and overfitting test

We observed that the Perceptron’s performance remained stable across both the training and testing sets, suggesting effective generalization. The mean AUC for the training set was 0.914 with a standard deviation (SD) of ±0.0153, while the validation set showed a mean AUC of 0.907 with an SD of ±0.0477. A two-sample t-test was applied to assess the statistical significance of the difference between these two sets, resulting in a value of p of 0.295. This non-significant value of p indicates that there is no substantial difference in the model’s performance on the training and testing sets, thus supporting the conclusion that overfitting is unlikely in our model.

This consistent performance across different subsets demonstrates the model’s reliability and its potential applicability in real-world scenarios where data variability is a common challenge.

#### 3.5.3 Input–output examples

In this chapter, we present examples of true positives, false negatives, true negatives, and false positives (Table 9), using input data and the output of the Perceptron. The logsig output of the Perceptron ranges between 0 and 1, where 0 indicates the presence of amblyopia or a risk factor condition, and 1 signifies the absence of the eye condition. Generally, the threshold for making a binary decision is set at 0.5. For finer adjustment, this threshold can be modified based on the ROC curve.

**Table 9:** Input-output examples.

**True negatives**		**Example #1**	**Example #1**	**Example #1**	**Example #1**	**Example #1**
INPUT	SRDS 8%	5	5	5	5	5
	DRDS 1%	4	3	4	3	5
	DRDS 0.7%	5	3	4	4	5
	DRDS 1%+0.5% noise	4	2	4	3	5
	Perceptron’s logsig OUTPUT	0.788	0.559	0.751	0.602	0.881
False positives		**Example #1**	**Example #1**			
INPUT	SRDS 8%	5	5			
	DRDS 1%	1	3			
	DRDS 0.7%	3	1			
	DRDS 1%+0.5% noise	1	1			
	Perceptron’s logsig OUTPUT	0.236	0.464			
True positives		**Example #1**	**Example #1**	**Example #1**	**Example #1**	**Example #1**
INPUT	SRDS 8%	1	1	5	5	5
	DRDS 1%	1	2	2	2	2
	DRDS 0.7%	0	2	4	1	2
	DRDS 1%+0.5% noise	1	3	1	2	4
	Perceptron’s logsig OUTPUT	<0.001	<0.001	0.438	0.290	0.321
False negatives		**Example #1**	**Example #1**	**Example #1**	**Example #1**	**Example #1**
INPUT	SRDS 8%	5	5	5	5	5
	DRDS 1%	5	5	4	5	3
	DRDS 0.7%	5	4	4	4	5
	DRDS 1%+0.5% noise	4	4	3	4	3
	Perceptron’s logsig OUTPUT	0.884	0.861	0.757	0.861	0.650

True negative examples were observed among emmetropic controls. Forty percent of these controls achieved the highest score, meaning they reached the maximum score (5) on each type of non-stereoacuity-based stereovision test (see last column). False positive examples from emmetropic controls were rare. These score patterns were more significant for amblyogenic conditions. True positive examples were identified among children with eye conditions. These examples highlight that, in some cases, while the static stereovision test was performed adequately, the dynamic tests frequently did not achieve satisfactory results, particularly in amblyogenic conditions. False negative examples from the amblyopic or amblyogenic group included only one amblyope who passed the test with refractive correction (see first column). However, without refractive correction, the child failed to pass (see last column of true positive examples). Some amblyogenic risk factors did not appear to lead to binocular abnormalities. These cases remain those of the unrecognized individuals by this method.

## 4 Discussion

In this study, we evaluated the performance of a new type of stereovision test called EuvisionTab Stereovision tests (ETS) in detecting amblyopia, amblyogenic and non-amblyogenic conditions in children. These tests were compared with four established clinical stereovision tests (Lang II, TNO, Stereo Fly, Frisby). ETS tests are distinct in that they: 1) do not rely on measuring stereoacuity; 2) can be either static or dynamic; 3) have low dot density; 4) can include uncorrelated noise, and 5) use artificial intelligence technology. Our results were supported by various statistical models, including AUC (DeLong’s method), matched-pair (McNemar’s exact), and non-matched (Fisher’s exact) tests. The sensitivity of ETS tests was found to be significantly better or equal to that of the most widely used clinical stereovision tests. The best-performing AI-based combination (AI-aw WC) was found to be more effective in detecting amblyopia or amblyogenic conditions than any of the classic stereovision tests.

### 4.1 Interpretation of the results

In this section, we summarize the advantages of novel, low-density, static and dynamic stereograms in vision screening compared to classic stereoacuity-based testing. Our results from ROC curve analysis, McNemar’s matched-pair test, and Fisher’s non-matched tests were consistent.

The AI-based tests showed significantly higher AUCs for detecting amblyopia (0.97–0.98) and amblyogenic conditions (0.81–0.84) compared to classic tests (0.82–0.95 for amblyopia, 0.6–0.74 for amblyogenic conditions). Sensitivity figures at optimum ROC points were higher for the novel tests (0.74–0.83) with AI-based tests showing high sensitivity in detecting amblyopia (0.96). Optimizing stimulus parameters and combining test results improved the specificities of the ETSs (0.87–0.98) to be comparable to classic tests (0.86–0.99).

Results from McNemar’s test showed that the AI test optimized for all conditions (AI-aw WC) outperformed most classic tests in detecting amblyopia or amblyogenic conditions. Fisher’s exact test revealed that the AI-aw WC test had significantly higher sensitivity in detecting amblyopia or an amblyogenic condition compared to TNO, while specificities did not differ significantly.

The study demonstrated that ETSs without refractive correction, typical in community screenings, were highly effective in detecting amblyopia. AI-based tests showed AUCs of 0.997 without refractive correction, indicating that they could identify all amblyopic individuals. This is a significant advantage in community screenings.

AI-based stereovision tests significantly outperformed classic tests in detecting amblyopia and amblyogenic conditions, with superiority evident when the goal is to detect amblyogenic conditions along with amblyopia. ETSs offer benefits for mass screening over traditional clinical tests, including simpler testing procedures, no monocular cues (38), unambiguous pass/fail decisions, flexibility in pass level adjustment, popularity among children, potential for AI optimization, suitability for telemedicine and home screening, and easy integration with patient data management systems.

### 4.2 Strengths and limitations of the study

#### 4.2.1 Strengths

The novel test under evaluation is robust and straightforward to administer. Its design is so user-friendly that even a child can self-administer the test (Figure 1). Moreover, the interactive nature of the test, use of mobile technology makes it engaging for children.

In this study, we conducted a comprehensive comparison of the new test with several well-established clinical stereovision tests. These classic tests were carefully selected to represent a broad range of methodologies, including random dot stereograms with different channel separations, real depth, and contour stereograms. We refrained from using predefined stereoacuity thresholds. Instead, we employed sophisticated ROC curve analysis to identify optimal cut-off points for binary classification. Multiple statistical methods were used, and they all yielded consistent results when comparing the novel test to classic tests.

#### 4.2.2 Limitations

Despite the strengths of the study, some limitations exist. Firstly, not all subjects underwent every test in a systematic manner. For a detailed explanation, please refer to the “Study Design” subsection within the Methods section. Secondly, the study included only a limited number of patients who were newly diagnosed with eye conditions. To emulate a setting that is representative of a typical screening environment, we tested participants without corrective lenses (“no correction” or NC test conditions). However, this was only possible with the new type of test. Furthermore, the age range of our participants was broader than the standard target demographic for amblyopia screening, which is typically 3.5 to 6 years.

### 4.3 Future of AI-based screening

This trial sought to identify optimal random dot stereogram parameters for screening amblyopia and amblyogenic conditions. During analysis, the idea of AI first emerged as a simple summation of ETS scores significantly improved AUC, suggesting potential for further optimization. Although the current study utilized a simplistic AI approach (perceptron) suitable for the dataset size, employing more complex AI solutions like multilayer feedforward backpropagation (39) or deep learning could enhance results in the future studies. Further exploration of RDS parameters, which could include other versions of stereograms with fewer repetitions, could also be beneficial.

Our study represents a significant advancement in vision screening, overcoming some limitations of traditional methods. We have developed an AI model that merges different types of stereograms, such as static, dynamic, and noisy, outperforming standard stereoacuity-based tests in identifying childhood vision impairments. Unlike typical AI applications that analyze facial images and eye positions to detect signs of amblyopia (40–43), our approach integrates multiple non-stereoacuity tests. This method effectively identifies all cases of amblyopia and its risk factors, not just those with visible symptoms. Additionally, it offers a more affordable solution for vision screening in areas with limited resources, moving away from expensive technologies like autorefraction and retina scanners.

## Data availability statement

The raw data supporting the conclusions of this article will be made available by the authors, without undue reservation.

## Ethics statement

The studies involving humans were approved by Regional Etical Board, University of Pécs. The studies were conducted in accordance with the local legislation and institutional requirements. Written informed consent for participation in this study was provided by the participants’ legal guardians/next of kin. Written informed consent was obtained from the minor(s)’ legal guardian/next of kin for the publication of any potentially identifiable images or data included in this article.

## Author contributions

ZC: Conceptualization, Visualization, Writing – original draft, Writing – review & editing, Data curation, Formal analysis, Investigation, Methodology, Project administration. EM-B: Conceptualization, Data curation, Formal analysis, Investigation, Methodology, Project administration, Supervision, Visualization, Writing – original draft, Writing – review & editing. AB: Conceptualization, Data curation, Investigation, Methodology, Project administration, Validation, Writing – review & editing. ABF: Formal analysis, Methodology, Software Writing – original draft, Writing – review & editing. ÁP: Data curation, Formal analysis, Methodology, Project administration, Writing – review & editing. VAN: Conceptualization, Data curation, Investigation, Project administration, Supervision, Writing – original draft, Writing – review & editing. LZ: Data curation, Formal analysis, Methodology, Software, Visualization, Writing – review & editing. DF: Formal analysis, Investigation, Methodology, Project administration, Software, Visualization, Writing – review & editing. AC: Conceptualization, Formal analysis, Methodology, Software, Writing – review & editing. KS-G: Data curation, Investigation, Methodology, Project administration, Writing – review & editing. PB: Funding acquisition, Resources, Software, Supervision, Writing – review & editing. DP: Conceptualization, Data curation, Investigation, Methodology, Project administration, Supervision, Writing – original draft, Writing – review & editing. GJ: Conceptualization, Funding acquisition, Software, Supervision, Visualization, Writing – original draft, Writing – review & editing, Data curation, Formal analysis, Methodology, Project administration, Resources, Validation.
