# Supplementary material for: Artificial intelligence-based screening for amblyopia and its risk factors: comparison with four classic stereovision tests
Source: Front Med (Lausanne). 2023 Dec 22;10:1294559. doi: 10.3389/fmed.2023.1294559 (PMC10775855; doi:10.3389/fmed.2023.1294559)
Supplement: Supplementary file 1 [file Data_Sheet_1.pdf]

SUPPLEMENTARY METHODS

1. Sensitivity, Specificity and Their Variance

Our goal is to compute a confidence interval for sensitivity and specificity of certain contingency tables. Using the usual language of positive/negative outcomes, a standard table looks like this (contingency-type table with relative frequencies instead of frequencies):

		Supposed outcome	
		positive	negative
Actual outcome	positive	p_{11}	p_{12}
	negative	p_{21}	p_{22}

Here p_{11} is an estimate of the probability of a true positive outcome, p_{12} is an estimate of the probability of a false positive outcome, p_{21} corresponds to an estimate of the probability of a false negative one and finally p_{22} gives us an estimate of the probability of a true negative outcome.

We are going to denote by π_{ij} the corresponding probabilities.

According to the definition, an estimate of the sensitivity for a contingency table (sensitivity for short) is given by:

$$\frac{p_{11}}{p_{11} + p_{21}} = \frac{p_{11}}{p_{+1}} = \hat{\theta}_1,$$

where we've adopted the notation: p_{+1} for the following sum: $p_{+1} = p_{11} + p_{21}$. Similarly p_{1+} would denote the sum $p_{1+} = p_{11} + p_{12}$.

For the observed sensitivity we are going to use $\theta_1 = \frac{\pi_{11}}{\pi_{+1}}$, where we used the same kind of summation short hand, as before.

An estimate of the specificity for a contingency table (specificity for short) is given by:

$$\frac{p_{22}}{p_{12} + p_{22}} = \frac{p_{22}}{p_{+2}} = \hat{\theta}_2,$$

using a similar notation to the one mentioned above.

In this case, the observed specificity will be denoted by $\theta_2 = \frac{\pi_{22}}{\pi_{+2}}$.

Date: December 12, 2023.

In order to compute a confidence interval for sensitivity lets reconsider the contingency table we had before: Instead of relative frequencies, we will use the actual outcomes of the experiments.

		Supposed outcome	
		positive	negative
Actual outcome	positive	N_{11}	N_{12}
	negative	N_{21}	N_{22}

Let $N = N_{11} + N_{12} + N_{21} + N_{22}$ the number of experiments we had. The standard approach to finding the confidence intervals for random variables with binomial distribution is the one proposed by C.J. Clopper and E.S. Pearson [10]. The method they proposed is termed "exact" because it tends to be conservative in estimating the interval. It is not "exact" in the sense of giving explicite values for the endpoints of the interval in question, except in the extreme cases.

We are going to consider sensitivity, but exactly the same method works for specificity. In case of sensitivity, the overall number of experiments is $N' = N_{11} + N_{21}$. Using binomial random variable for the model, we have N_{11} successes and N_{21} failures and hence we have an estimate for the probability of success: $\frac{N_{11}}{N'}$.

Suppose we would like to cover $100(1-\alpha)\%$ of the cases in a two-sided fashion, where α is a prescribed value. For example, if we want to have 95% coverage we would use $\alpha = 0.05$.

In order the find a confidence interval round our estimate we need to ask the following questions. First: What is the smallest $p = p_L$ such that the probability of seeing N_{11} or more successes is $\alpha/2$. Second: What is the largest $p = p_U$ such that the probability of seeing N_{11} or fewer successes is $\alpha/2$. In other words, the probability of seeing $N_{11}+1$ or more successes is $1-\alpha/2$.

In general, p_L and p_U can be found only numerically. One way to do this is by inverting the β distribution. Suppose Y is a random variable with βdistribution whose shape parameters are N_{11} for the first one and $N' - N_{11} + 1$ for the second one. Then p_L is the value for which the probability $\mathbb{P}(Y < p_L) = \alpha/2$. Now, suppose Y is a random variable with βdistribution whose shape parameters are $N_{11}+1$ for the first one and $N' - N_{11}$ for the second one. Then p_U is the value for which the probability $\mathbb{P}(Y < p_U) = 1 - \alpha/2$.

The corresponding confidence interval is (p_L, p_U).

2. Comparison of Diagnostic Tests

2.1. **McNemar's Matched Pairs.** Suppose we have two (or more) tests that were administered to the same cohort and we would like to compare the accuracy of those two tests. By accuracy of a test we mean the number of correctly (by the test) identified instances (true positives plus true negatives plus false positives) divided by the number of tests performed. In order to do this we need to consider the entire cohort of subjects. We need to know how many subject passed both tests (n_{11}), how many passed the first one but failed the second one (n_{12}), how many passed the second one but failed the first one (n_{21}) and finally, how many failed both (n_{22}). The resulting table looks like this:

		2nd Test		
		pass	fail	
1st Test	pass	n_{11}	n_{12}	$n_{11}+n_{12}$
	fail	n_{21}	n_{22}	$n_{21}+n_{22}$
		$n_{11}+n_{21}$	$n_{12}+n_{22}$	n

The first test is a diagnostic test, while the second test could be either another diagnostic test or the gold standard.

The size of the cohort is denoted by n and it is $n_{11}+n_{12}+n_{21}+n_{22}$. By definition, the estimator for the accuracy of the first test is

$$\widehat{acc}_1 = \frac{n_{11}+n_{12}+n_{22}}{n},$$

and for the second test, it is

$$\widehat{acc}_2 = \frac{n_{11}+n_{21}+n_{22}}{n},$$

so the difference is

$$\widehat{acc}_1 - \widehat{acc}_2 = \frac{n_{12}-n_{21}}{n}.$$

Following [3] and [4] we introduce a random variable X, such that X measures the disagreement between the two tests: It takes on the value 0 if they agree, it takes on the value 1 if, say, the subject passed the first test but did not the second one, and it takes on the value -1 if it is the other way around. The estimator of the expectation of X

is $\dfrac{n_{12}-n_{21}}{n}$, which is exactly $\widehat{acc}_1 - \widehat{acc}_2$. A simple calculation shows that the estimator of the variance of X is

$$\sigma^2 = \frac{1}{n^2}\left(n\left(n_{12}+n_{21}\right) - \left(n_{12}-n_{21}\right)^2\right).$$

The null-hypothesis is that the two accuracies are the same, so in this case

$$\sigma^2 = \frac{n_{12}+n_{21}}{n}.$$

We need the standard error of the difference between the accuracies. In general, if Y is a random variable and σ_Y^2 is its variance, while we performed n experiments, the estimate for the standard error of Y is

$$\widehat{se}(Y) = \frac{\sigma_Y^2}{\sqrt{n}}.$$

In our case, we need the standard error of $\widehat{acc}_1 - \widehat{acc}_2$, the difference between the accuracies:

$$\widehat{se}\left(\widehat{acc}_1 - \widehat{acc}_2\right) = \frac{\sigma}{\sqrt{n}} = \frac{\sqrt{n_{12}+n_{21}}}{n}.$$

McNemar's test is a chi-square test (asymptotically, with 1 df, see [4]) which has the following form:

$$\chi^2 = \left(\frac{|\widehat{acc}_1 - \widehat{acc}_2| - \frac{1}{n}}{\widehat{se}\left(\widehat{acc}_1 - \widehat{acc}_2\right)}\right)^2 = \frac{\left(|n_{12}-n_{21}|-1\right)^2}{n_{12}+n_{21}}.$$

If the value of χ^2 is large we can infere that the sensitivity of the tests are different.

In case we don't have too much data, we can instead use an exact test, the exact binomial test, in order to compute an exact two-sided P value. Suppose $n_{12} \geq n_{21}$ (of course it works the other way around, too, but requires more calculation). The exact two-sided P value is

$$P = 2\sum_{k=n_{12}}^{n_{12}+n_{21}} \binom{n_{12}+n_{21}}{k}\left(\frac{1}{2}\right)^{n_{12}+n_{21}}$$

$$= \left(\frac{1}{2}\right)^{n_{12}+n_{21}-1} \cdot \sum_{k=n_{12}}^{n_{12}+n_{21}} \binom{n_{12}+n_{21}}{k}.$$

2.2. **Fisher's Exact Test.** We can use Fisher's Exact Test [1, 2] to compare the sensitivities and specificities of given experiments. Suppose we have a certain number of examinations using multiple diagnostic tests and we would like to compare the sensitivity of the kth test with the lth test. Consider the following contingency table:

	kth diagnostic test	lth diagnostic test	Row total
True Positive	n_{11}	n_{12}	$n_{11} + n_{12}$
False Negative	n_{21}	n_{22}	$n_{21} + n_{22}$
Column total	$n_{11} + n_{21}$	$n_{12} + n_{22}$	

In general the probability that we will see exactly this arrangement in the table, if the marginals are given, can be described by a multinomial distribution. However, if our null-hypothesis H_0 is that the sensitivity of the kth test is the same as the sensitivity of the lth test, then the probability P that this particular arrangement occurs is described using a hypergeometric distribution:

$$P = \frac{\binom{n_{11}+n_{12}}{n_{11}}\binom{n_{21}+n_{22}}{n_{21}}}{\binom{n_{11}+n_{12}+n_{21}+n_{22}}{n_{11}+n_{21}}} = f(n_{11}; n, n_{1+}, n_{+1}),$$

where $n = n_{11}+n_{12}+n_{21}+n_{22}$. Here we used the same notation as above and denoted the marginals by $n_{1+} := n_{11} + n_{12}$, while $n_{+1} := n_{11} + n_{21}$. Since n, n_{1+} and n_{+1} are fixed, the value of P depends only on one parameter. The smaller the P value the more evidence we have against the null-hypothesis.

If the alternative hypothesis H_1 is the one-sided hypothesis that the sensitivity of the kth test is larger than the sensitivity of the lth test, then the corresponding p-value (significance) can be calculated exactly:

$$p = \sum_{t \in S} f(t; n, n_{1+}, n_{+1}),$$

where S is the set of all integers that are larger than or equal to n_{11} and the corresponding arrangement gives us the prescribed marginals. Note, that since n, n_{1+} and n_{+1} are prescribed, one parameter determines the shape of the whole table.

There is more than one method to test against a two-sided alternative hypothesis and it seems that the choice is a matter of taste. See [1] for more details.

Suppose we had access to a gold standard, i.e. an omniscient observer, who would know exactly which tests actually failed and which

	diagnostic test	gold standard	Row total
True Positive	N_{11}	$N_{11} + N_{21}$	$2N_{11} + N_{21}$
False Negative	N_{21}	0	N_{21}
Column total	$N_{11} + N_{21}$	$N_{11} + N_{21}$	

tests passed. In order to describe such an observer using the terminology of false/true positives and false/true negatives, we can do the following: Suppose the real world observer sees N_{11} true positive outcomes, N_{21} false negatives, N_{12} false positives and finally N_{22} true negatives. Then the ideal observer would have observed $N_{11} + N_{21}$ true positives, 0 false negatives, 0 false positives and $N_{12} + N_{22}$ true negatives.

This means, that if we want to use the Fisher exact test to compare the sensitivity of a real live test with the gold standard we would compute the following:

$$P = \frac{\binom{N_{11}+N_{11}+N_{21}}{N_{11}}\binom{N_{21}}{N_{21}}}{\binom{N_{11}+N_{11}+N_{21}+N_{21}+0}{N_{11}+N_{21}}} = \frac{\binom{2N_{11}+N_{21}}{N_{11}}}{\binom{2(N_{11}+N_{21})}{N_{11}+N_{21}}}.$$

A same kind of computation can be done for specificity.

3. Corrections for Multiple Comparisons

3.1. **Bonferroni Correction.** Suppose we have multiple hypotheses, for example because we are comparing multiple tests pairwise but at the same time. Suppose we have, say m hypotheses — some of them true, others false. Let α be treshold value for the probability that we reject at least one true hypothesis. We would accept the collection of hypotheses if the familywise error rate, i.e. the probability of rejecting at least one true hypothesis, is below α. The Bonferroni correction method [5] says that we should test each hypothesis at level $\frac{\alpha}{m}$. If each hypothesis test passes at this level then the probability that we reject at least one true hypothesis is not more than α. This result is a direct consequence of the simplest Bonferroni inequality.

3.2. **False-discovery Rate.** Besides the Bonferroni-correction, there are other methods to test multiple hypotheses at once. One such method looks at the False-discovery Rate (see, for example [6]). Suppose we have m hypotheses and m_0 of them happen to be true, where $0 \leq m_0 \leq m$. Suppose we have R rejections out of m tests. Let V be the number of occasions when the null hypothesis was true but it

was rejected (i.e. the number of false discoveries). The false-discovery proportion is $\frac{V}{R}$ if $R > 0$ and 0 is $R = 0$. The false-discovery rate is defined as the expectation of the false-discovery proportion.

If we would like to control the false-discovery rate at level α we can use the Benjamini and Hochberg method or algorithm [9]. Suppose the tests applied produce p-values: p_i is the p-value associated with the ith test. Let $\left(p_{(i)}\right)_i$ be an ordered list of these p-values: $p_{(i)}$ is the p-value that happens to occupy the ith position of the ordered list. The algorithm says that we should find the largest index i_{max} such that $p_{(i_{max})}$ is less than a certain treshold value (specified below) and reject every hypothesis with p-value less than or equal to $p_{(i_{max})}$ [5, 7, 8]. If the p-values are independent then the threshold value is

$$t_i = \frac{i\alpha}{m},$$

while in general (without assuming independence) it is

$$t_i = \frac{i\alpha}{mC_m},$$

where $C_m = \sum_{i=1}^{m} \frac{1}{i}$. In both cases

$$i_{max} = \max \left\{ i : p_{(i)} < t_i \right\}.$$

According to the Benjamini and Hochberg algorithm if p_i (the p-value associated to the ith hypothesis) is less than $p_{(i_{max})}$ (the p-value from the ordered list identified above) then we should reject the ith null-hypothesis.

Graphically this means that we superimpose a straight line with slope $\frac{\alpha}{m}$ (or $\frac{\alpha}{mC_m}$ in general) over the plot of the ordered p-values and find the last (or largest) index where the p-value-plot dips under the straight line. That will be our i_{max}.

References

[1] Alan Agresti, *A survey of exact inference for contingency tables*, Statistical science **7** (1992), no. 1, 131–153.

[2] Erich L Lehmann and Joseph P Romano, *Testing statistical hypotheses*, Springer Science & Business Media, 2006.

[3] Quinn McNemar, *Note on the sampling error of the difference between correlated proportions or percentages*, Psychometrika **12** (1947), no. 2, 153–157.

[4] Joseph L Fleiss, Bruce Levin, and Myunghee Cho Paik, *Statistical methods for rates and proportions*, John Wiley & Sons, 2013.

[5] Jelle J. Goeman and Aldo Solari, *Multiple hypothesis testing in genomics*, Statistics in medicine **33** (2014), no. 11, 1946–1978.

[6] Anirban DasGupta, *Asymptotic theory of statistics and probability*, Springer Science & Business Media, 2008.

[7] Larry Wasserman, *All of statistics: a concise course in statistical inference*, Vol. 26, Springer, 2004.

[8] Trevor Hastie, Robert Tibshirani, and Jerome Friedman, *The elements of statistical learning: Data mining, inference, and prediction*, Springer, 2009.

[9] Yoav Benjamini and Yosef Hochberg, *Controlling the False Discovery Rate: A Practical and Powerful Approach to Multiple Testing*, Journal of the Royal Statistical Society. Series B (Methodological) **57** (1995), no. 1, 289–300.

[10] Charles J Clopper and Egon S Pearson, *The use of confidence or fiducial limits illustrated in the case of the binomial*, Biometrika **26** (1934), no. 4, 404–413.
