# Supplementary material for: Artificial intelligence-based screening for amblyopia and its risk factors: comparison with four classic stereovision tests
Source: Front Med (Lausanne). 2023 Dec 22;10:1294559. doi: 10.3389/fmed.2023.1294559 (PMC10775855; doi:10.3389/fmed.2023.1294559)
Supplement: Supplementary file 2 [file Data_Sheet_2.PDF]

Specificity at fixed sensitivity

Test name	Amblyopia			Amblyogenic			Nonamblyogenic		
	Sensitivity			Sensitivity			Sensitivity		
	0.90	0.95	Actual	0.90	0.95	Actual	0.90	0.95	Actual
SRDS 8 NC	0.60	0.30	-	0.16	0.08	-	0.09	0.02	-
DRDS 1 NC	0.85	0.68	-	0.15	0.07	-	0.09	0.06	-
DRDS 0.7 NC	0.93	0.90	-	0.68	0.38	-	0.15	0.05	-
DRDS 1+noise NC	0.79	0.69	-	0.59	0.33	-	0.15	0.08	-
AI-sum NC	0.97	0.96	-	0.64	0.43	-	0.16	0.08	-
AI-w NC	0.98	0.98	-	0.60	0.48	-	0.16	0.08	-
AI-aw NC	0.98	0.98	-	0.56	0.41	-	0.16	0.08	-
SRDS 8 WC	0.50	0.25	-	0.14	0.07	-	0.10	0.05	-
DRDS 1 WC	0.58	0.31	-	0.14	0.07	-	0.10	0.05	-
DRDS 0.7 WC	0.80	0.68	-	0.17	0.09	-	0.12	0.06	-
DRDS 1+noise WC	0.79	0.69	-	0.19	0.09	-	0.11	0.06	-
AI-sum WC	0.96	0.91	-	0.43	0.23	-	0.16	0.08	-
AI-w WC	0.98	0.98	-	0.41	0.23	-	0.16	0.08	-
AI-aw WC	0.98	0.98	-	0.50	0.23	-	0.16	0.08	-
Lang II	<0.99		0.65	<0.99		0.22	0.99		0.05
TNO	0.86	0.74	-	<0.68		0.58	<0.68		0.35
Stereo Fly	0.70	0.37	-	0.035	0.035	-	0.035	0.035	-
Frisby	<0.86		0.76	<0.86		0.44	<0.86		0.17

Table S1 *Specificity of stereo tests with sensitivity set to 0.90 and 0.95. NC: no correction; WC: with correction; SRDS 8: 8% density static test; DRDS 1: 1% density dynamic test; DRDS 0.7: 0.7% density dynamic test; DRDS 1+noise: 1% density dynamic test with 0.5% binocularly uncorrelated noise; AI-ETS: artificial intelligence-based ETS tests; sum: equally weighted sum of the four ETS-tests; w: 'weight', optimized weight for amblyopia; aw: 'average weight', optimized weight for all pathologic conditions.*

Explanation of specificity at fixed sensitivity

An advanced method for comparing the performance of various classifiers involves estimating specificity at a fixed sensitivity or vice versa. Both approaches are equally effective for comparisons. In this study, we opted for the fixed sensitivity method. This choice was made because maintaining an acceptable level of fixed specificity required very low pass level scores for ETSs, contradicting Bernoulli's principle for trials. In other words, the test could have been easily passed by chance if specificity had been fixed at 0.90. Therefore, we fixed sensitivity at 0.90 and 0.95 and estimated specificity accordingly. Where sensitivity could not be set to 0.90, we specified the maximum achievable sensitivity in the actual sensitivity column. Our comparison shows that AI-ETSs outperformed other tests under all conditions. Dynamic ETSs performed similarly or better than static ones, and both the lowest density and noisy ETSs exceeded the performance of most classic tests, except for TNO.

Conclusion

When comparing specificities at fixed sensitivities of 0.9 or 0.95, AI-based tests demonstrate higher specificity both in amblyopia (ranging from 0.91 to 0.98) and amblyogenic conditions (ranging from 0.23 to 0.5). This indicates superior overall performance compared to classic tests, which show specificities of 0.37 to 0.86 in amblyopia and 0.035 in amblyogenic conditions, as shown in Table S1. Typically, increasing the test's difficulty by adjusting the threshold enhances

sensitivity at the expense of specificity. It appears that there is a sensitivity ceiling for classic, stereoacuity-based tests, as revealed by our study. In amblyogenic conditions, sensitivities of these tests cannot be increased beyond a certain point. This is also true for Lang and Frisby tests in amblyopia, except for the Stereo Fly. This limitation is likely due to the minimum measurable stereoacuity in these tests: 200”, 60”, and 85” for Lang, TNO, and Frisby, respectively. In contrast, the Stereo Fly can measure stereoacuity down to 40”, achieving a sensitivity of 0.95. However, this limitation does not apply to ETS-type stereovision tests, where sensitivity can reach 1.00 by adjusting the pass level.

Comparing performance of stereovision tests to ophthalmological gold standard

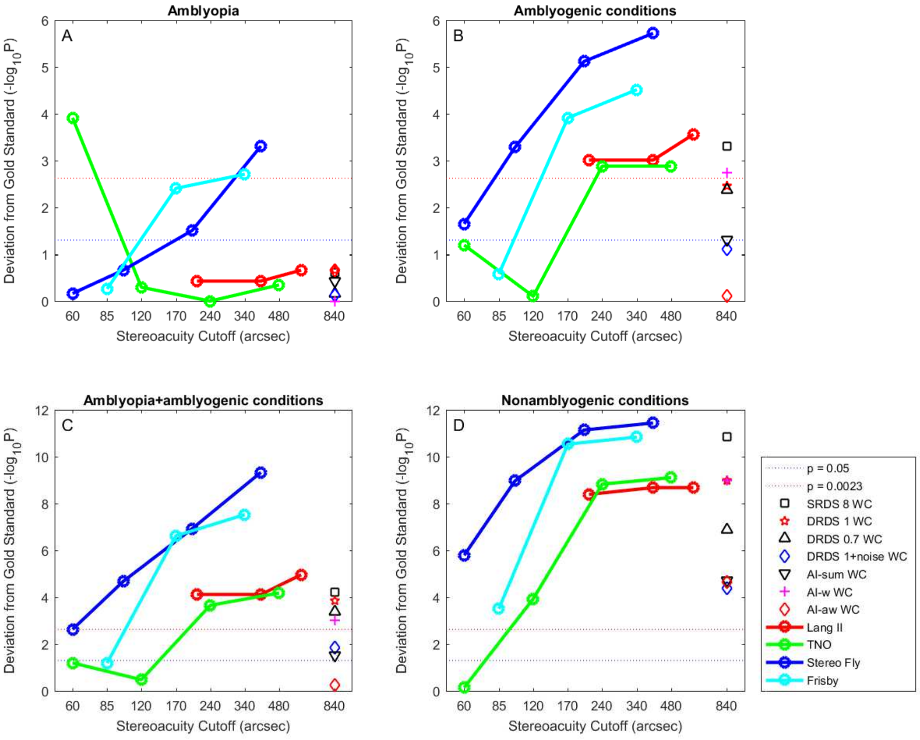

Figure S1: *Comparing stereovision test accuracy to the gold standard (GS).*
This figure illustrates how different stereovision tests align with the ophthalmologist's diagnosis, which we regard as the gold standard. Vertical Axis: Shows the negative logarithm of the p-value from McNemar's exact matched pair statistical test. This value measures deviation from the GS, with zero indicating minimal deviation. Higher values signify greater deviation. Horizontal Axis: Represents stereoacuity cutoff levels in arcseconds on a logarithmic scale for each stereoacuity-based test. The ETS results are at the far right, marked at 840". Lines: Dotted blue line indicates p=0.05; dotted red line represents the Bonferroni corrected p-value for multiple comparisons (n=21). Panels: Each panel corresponds to a specific cohort comprising a study group and controls. The study group is identified in the panel title. Abbreviations: Refer to Table 2 and 7 for abbreviations of stereo tests (e.g., SRDS 8, DRDS 1). Detailed explanations are in the results section.

Comparing stereovision tests to ophthalmological gold standard

A significantly higher AUC indicates improved overall classification performance of a test. We investigated whether various tests corresponded with the classification of ophthalmological diagnoses, our established "gold standard." This analysis, which required data on false positives and negatives, involved cohorts comprising members of specific study groups (such as amblyopes) and control participants.

Deviation from the gold standard was quantified as the negative logarithm of the p-value obtained from McNemar's test, which is used to reject the null hypothesis (detailed in Supplementary methods). Smaller p-values signify greater deviation from the gold standard; thus, higher values on the negative logarithm scale indicate more significant deviation, whereas values near zero suggest minimal deviation.

Since classical tests do not have predefined pass levels, we evaluated them across all possible stereoacuity thresholds. The balanced design of McNemar's test ensured unbiased results, eliminating the potential for skew due to varying data volumes from each test. In these statistical comparisons, only data from participants who underwent all tests were included (refer to Table 8).

Figure S1 illustrates the deviation of these tests from the gold standard at different stereoacuity cutoff levels for each study group. Since ETSs do not measure stereoacuity (having a fixed disparity of 840”), their deviation is represented at the extreme right on the abscissa. Each graph panel includes two dotted lines, in blue and red, indicating the conventional and Bonferroni-corrected rejection thresholds for the null hypothesis at p=0.05, respectively. For the Bonferroni correction, the value of 0.05 was divided by the number of data points in each panel.

Theoretical understanding suggests that setting the stereoacuity cutoff level very low increases false positives, while a very high cutoff raises false negatives, both scenarios leading to reduced accuracy. An optimal cutoff should exist where both false positives and negatives are minimized, thereby closely aligning with the gold standard.

This U-shaped pattern was evident in the amblyopia group (n=68, comprising 23 amblyopic and 45 control participants) using the TNO test, as shown in Fig S1A. For amblyopia detection, the optimal cutoff for TNO was identified at 240” aligning with both our expectations and existing literature (22,27). Setting the cutoff at the lowest level of 60” significantly reduced accuracy due to increased false positives and reduced specificity. Other classic tests demonstrated adequate accuracy in detecting amblyopia, except for TNO at 60”, Frisby at 340”, and Stereo Fly at 400”. All ETSs exhibited very good accuracy (p>0.05) compared to the gold standard in this group.

Performance of classic tests was notably poorer in the amblyogenic group (n=68, with 23 amblyogenic and 45 control participants), as seen in Figure S1B. The TNO test's minimum effective cutoff in the U-shaped curve shifted to 120”. Few tests remained below the p=0.05 line: TNO at 60” and 120”, Frisby at 85”, and three ETSs (AI-aw WC, AI-sum WC, and DRDS 1+noise WC). Post-Bonferroni correction, most ETSs closely matched the gold standard except for SRDS 8 WC and AI-w WC, while most classic tests at common cutoff levels did not.

In the combined amblyopia and amblyogenic group (n=91), only three classic tests and the AI-aw WC test closely matched the gold standard (Figure S1C). After applying Bonferroni's correction, AI-sum WC and DRDS-1+noise-WC remained close to the gold standard.

In the nonamblyogenic group (n=84, with 39 nonamblyogenic and 45 control participants), the only test with sufficient accuracy was the TNO at a 60" cutoff (Figure S1D). This is possibly due to the TNO's small random dot size, which might allow refractive issues to influence results at this low threshold.

Figure S1C indicates that in the amblyopia+amblyogenic condition, the AI-aw WC version of the ETS test performed best. Further analysis tested whether AI-aw WC significantly outperformed other tests. Multiple pairwise comparisons via McNemar's test (with Benjamini-Hochberg correction) revealed that for detecting amblyopia, AI-aw WC was significantly more effective than several other tests, except for a few specific conditions. In amblyogenic conditions, AI-aw WC outperformed all tests barring TNO at 120", Frisby at 85", and Stereo Fly at 60". In the amblyopia+amblyogenic condition, only TNO at 120" and Frisby at 85" were on par with AI-aw WC, while the rest fell short.

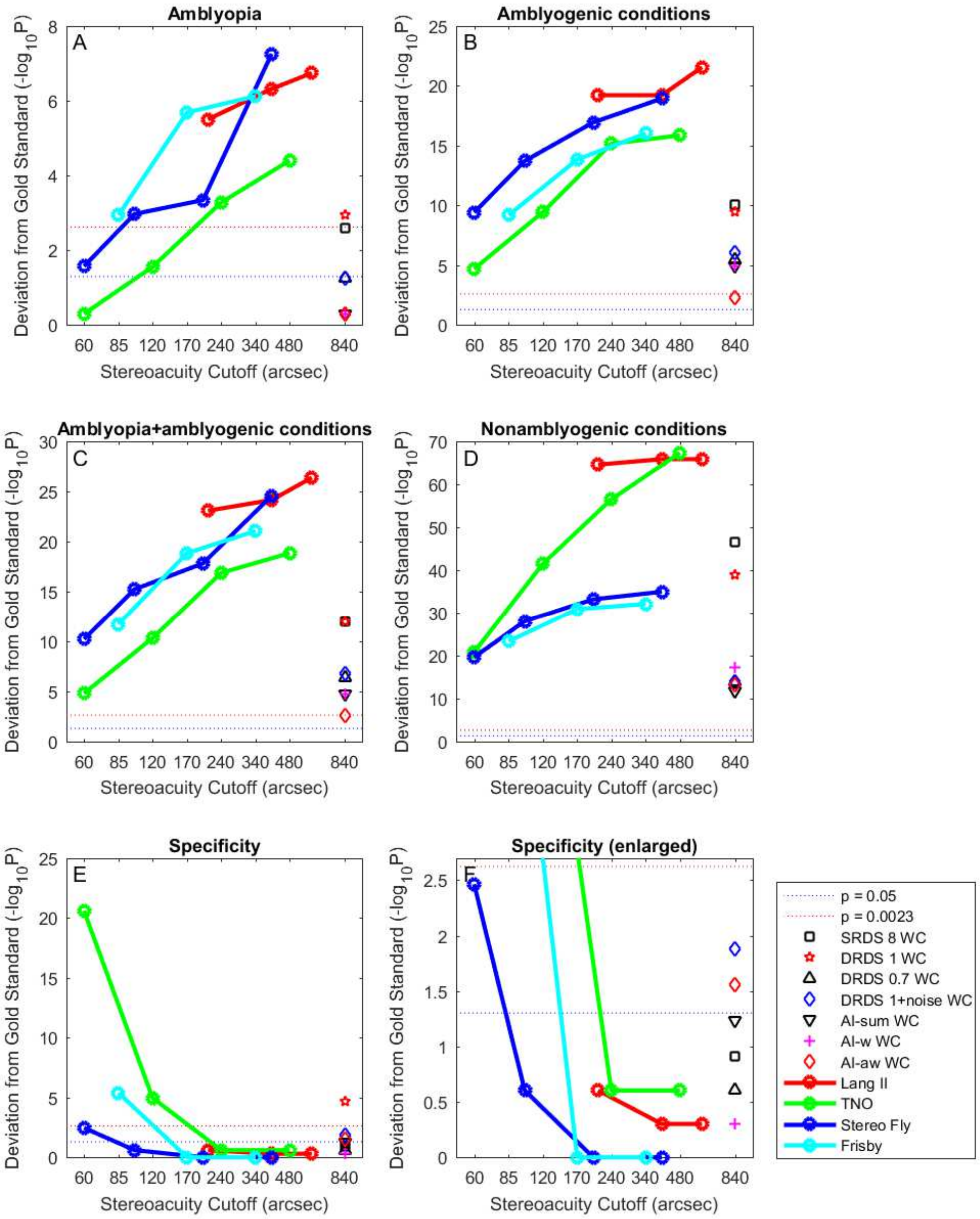

***Figure S2**: Comparison of stereovision test sensitivities and specificities against the gold standard.*
The vertical axis represents the negative logarithm of the p-value obtained from Fisher's exact statistical test, which was used to compare the results of binary classification against the ophthalmologist's diagnosis, accepted as the gold standard (GS). A value of zero indicates no or minor deviation from the GS, with higher numbers indicating larger deviations. The horizontal axis depicts the stereoacuity cutoff level in arcseconds on a logarithmic scale, used for the stereoacuity-based classic tests. Results for the ETSs are plotted at the far right, positioned at 840". The dotted blue line represents a p-value of 0.05, while the dotted red line indicates the Bonferroni-corrected p-value for multiple comparisons, with n=21. Panels A-D display data for one study group each, as indicated in their respective titles. Panels E and F present classification results for the control group, with the left (E) and right (F) panels showing the

same data but with two different vertical scalings. The abbreviations for the various stereo tests, consistent with those in Fig S1, are listed in the legend and can also be found in Tables 2 and 7.

Comparison of sensitivities and specificities to the „gold standard"

In previous analyses, the sensitivity and specificity of a test could compensate for each other. To further delve into this, we compared the sensitivities of various stereovision tests using Fisher's exact non-parametric statistics against the ophthalmologists' diagnosis, considered the gold standard. This analysis, focusing on the study groups and excluding controls, is depicted in Figure S2 for sensitivities. Here, only study groups (participants with the specified condition) were included. The comparison method, accuracy measures, study groups, and panel arrangement mirror those in Fig S1. Theoretically, as the stereoacuity cutoff level increases, sensitivities decrease. This decrease, observed as a deviation from the gold standard, is evident in each panel, regardless of the study group.

For the amblyopia group (Fig S2A), at first glance, the sensitivities of ETSs appear higher than those of classic stereovision tests. Most ETSs remained below the p=0.05 line (exceptions being SRDS 8 WC and DRDS 1 WC), indicating no significant difference from the gold standard. Conversely, most classic tests at various stereoacuity cutoff levels (except TNO at 60") significantly deviated from the gold standard ($p<0.05$). Applying Bonferroni's correction, only Stereo Fly at 60" and TNO at 60" and 120" remained below the red dotted line ($p>0.0023$), suggesting no significant difference from the gold standard.

In direct pairwise comparisons by Fisher's test, the sensitivity of AI-aw WC was not significantly different from Frisby at 85", TNO at 60", TNO at 120", and Stereo Fly at 60". However, AI-aw WC significantly outperformed TNO at 240" and all other data points above it ($p<0.05$, Benjamini-Hochberg method). AI-w WC and AI-sum WC, overlapping with AI-aw WC, showed similar results.

In the amblyogenic condition group (Fig S2B), no tests stayed below the p=0.05 threshold, indicating all fell short of the gold standard. AI-aw WC alone remained under the Bonferroni corrected threshold, which is not significant on its own. Fisher's test revealed AI-aw WC did not differ significantly from TNO at 60", but it did from all other data points, including TNO at 120" ($p<0.05$, Benjamini-Hochberg method). The other two AI tests showed no significant improvement over other tests except for Stereo Fly at 200" and 400" ($p<0.05$).

In the combined amblyopia and amblyogenic groups (Fig S2C), AI-aw WC significantly outperformed all classic tests at any stereoacuity cutoff level ($p<0.05$, Benjamini-Hochberg method), except TNO at 60". Notably, AI-w WC and AI-sum WC also differed significantly from most classic tests ($p<0.05$), except TNO at 120", Stereo Fly at 60", and Frisby at 85".

In the nonamblyogenic group (Fig S2D), all tests significantly fell short of the gold standard.

We then assessed the specificity of classifications against the gold standard (Fig S2E-F). For specificity calculations, which require the number of true negatives and false positives, only the control (emmetropic) dataset was used. The left and right panels present the same data with different vertical scalings. AI-aw WC's specificity significantly lagged behind all classic tests ($p<0.05$) except for Stereo Fly at 60”, Frisby at 85”, and TNO at 120”. In contrast, AI-w WC's specificity did not significantly differ from that of the classic tests. Both AI-aw WC and AI-w WC showed better specificity than TNO at 60” ($p<0.05$). AI-sum WC resembled AI-aw WC, except it did not significantly differ from Stereo Fly at 100”.

Conclusion

Since ophthalmologic diagnoses were obtained for each participant, we used them as a gold standard for comparing various stereovision tests. McNemar's statistical test revealed that most tests perform well in detecting amblyopia, but the AI test, optimized for all conditions (i.e., AI-aw WC), outperforms most classic tests in identifying the presence of amblyopia or an amblyogenic condition (see Fig. S1A-C). This result suggests a superior classification performance of dynamic ETSs over traditional stereovision tests in amblyogenic conditions, a finding further substantiated by subsequent analyses.

When sensitivities and specificities were compared separately using Fisher’s test, it was found that the AI-aw WC test demonstrated significantly higher sensitivity in detecting amblyopia or an amblyogenic condition (as shown in Fig. S2A-C) compared to any classic test. However, there were no significant differences in specificity between the AI-aw WC test and the classic tests (see Fig. S2E-F).
